# Focal brain lesions induced with ultraviolet irradiation

**DOI:** 10.1038/s41598-018-26117-w

**Published:** 2018-05-22

**Authors:** Mariko Nakata, Kazuaki Nagasaka, Masayuki Shimoda, Ichiro Takashima, Shinya Yamamoto

**Affiliations:** 1 Systems Neuroscience Group, Human Informatics Research Institute, National Institute of Advanced Industrial Science and Technology (AIST), 1-1-1 Umezono, Tsukuba, 305-8568 Japan; 2 Japan Society for Promotion of Science, 5-3-1 Koujimachi, Chiyoda-ku, Tokyo 102-0083 Japan; 3 Graduate School of Comprehensive Human Sciences, University of Tsukuba, 1-1-1 Tennodai, Tsukuba, 305-9577 Japan; 4 Department of Pathology, Keio University School of Medicine, 35 Shinanomachi, Shinjuku-ku, Tokyo 160-8582 Japan

## Abstract

Lesion and inactivation methods have played important roles in neuroscience studies. However, traditional techniques for creating a brain lesion are highly invasive, and control of lesion size and shape using these techniques is not easy. Here, we developed a novel method for creating a lesion on the cortical surface via 365 nm ultraviolet (UV) irradiation without breaking the dura mater. We demonstrated that 2.0 mWh UV irradiation, but not the same amount of non-UV light irradiation, induced an inverted bell-shaped lesion with neuronal loss and accumulation of glial cells. Moreover, the volume of the UV irradiation-induced lesion depended on the UV light exposure amount. We further succeeded in visualizing the lesioned site in a living animal using magnetic resonance imaging (MRI). Importantly, we also observed using an optical imaging technique that the spread of neural activation evoked by adjacent cortical stimulation disappeared only at the UV-irradiated site. In summary, UV irradiation can induce a focal brain lesion with a stable shape and size in a less invasive manner than traditional lesioning methods. This method is applicable to not only neuroscientific lesion experiments but also studies of the focal brain injury recovery process.

## Introduction

Creation of a focal brain lesion has been one of the most fundamental and essential techniques in the field of neuroscience. Traditionally, a targeted brain site has been destroyed via physical removal of the tissue (e.g., aspiration or cutting with a knife)^1–4^, application of an electrical current^5,6^, or injection of drugs^7,8^. However, these methods are highly invasive; i.e., they require direct access to the targeted site via a needle or an electrode, which breaks membranous tissues covering and protecting the brain (e.g., dura mater). Dura mater breakage may induce several problems in a long-term experiment, including infection or contamination, potentially introducing an artefact into the results. Moreover, prediction and/or control of the shape and size of a lesion using these techniques is often difficult because of uncertainties such as spillover of the drug. Thus, it is necessary to develop a novel experimental method for creating a focal brain lesion in a less invasive and more easily controllable manner.

To lesion a small targeted brain area, we intended to use light energy. A ray of light, especially light with a short wavelength, can destroy living tissue. In particular, ultraviolet (UV) light (wavelength 10~380 nm) possesses more energy than visible light. It is known that UV irradiation of living tissue (e.g., skin) induces several reactions, including production of reactive oxygen species (ROS), inflammation, and cell death. Nonetheless, the outcome of UV irradiation depends on the light wavelength and exposure amount^9–12^.

However, it is also known that a light ray travelling through an object is attenuated to an extent that depends on the characteristics of the object and the light wavelength. Light with a shorter wavelength possesses less penetration ability than light with a longer wavelength. For our objective of generating a lesion in cortical tissue without breaking the dura, it was necessary to choose a long enough wavelength to reach the brain tissue from above the dura. Thus, we used a UV light classified as UV-A. UV light is categorized into three classes according to wavelength: UV-A (320~380 nm), UV-B (280~320 nm), and UV-C (shorter than 280 nm); UV-A light penetrates more deeply into tissue than other UV-B or UV-C light^13^. Moreover, UV-A light damages not only DNA directly^14,15^ but also various cellular structures^12^. Therefore, we hypothesized that UV-A light can be applied for the creation of a focal brain lesion on the cerebral cortex.

In the present study, the brain surface of rodent model animals was exposed to UV-A light with a wavelength of 365 nm. We hypothesized that 365 nm UV irradiation would disrupt the cortical layer without breaking the dura because of its relatively high permeability. We examined the influence of UV irradiation from an optical fibre placed above the dura and explored the potential of utilizing UV irradiation as a novel experimental technique for creating a focal brain lesion. We further investigated whether size of the UV irradiation-induced brain lesion was controllable, whether the lesion could be visualized in a living animal, and whether generation of a lesion with this technique could disrupt neural activity and neural transmission at the irradiated site.

## Results

### UV irradiation over dura induced neuronal degeneration and glial accumulation at the brain surface

**Figure 1:**
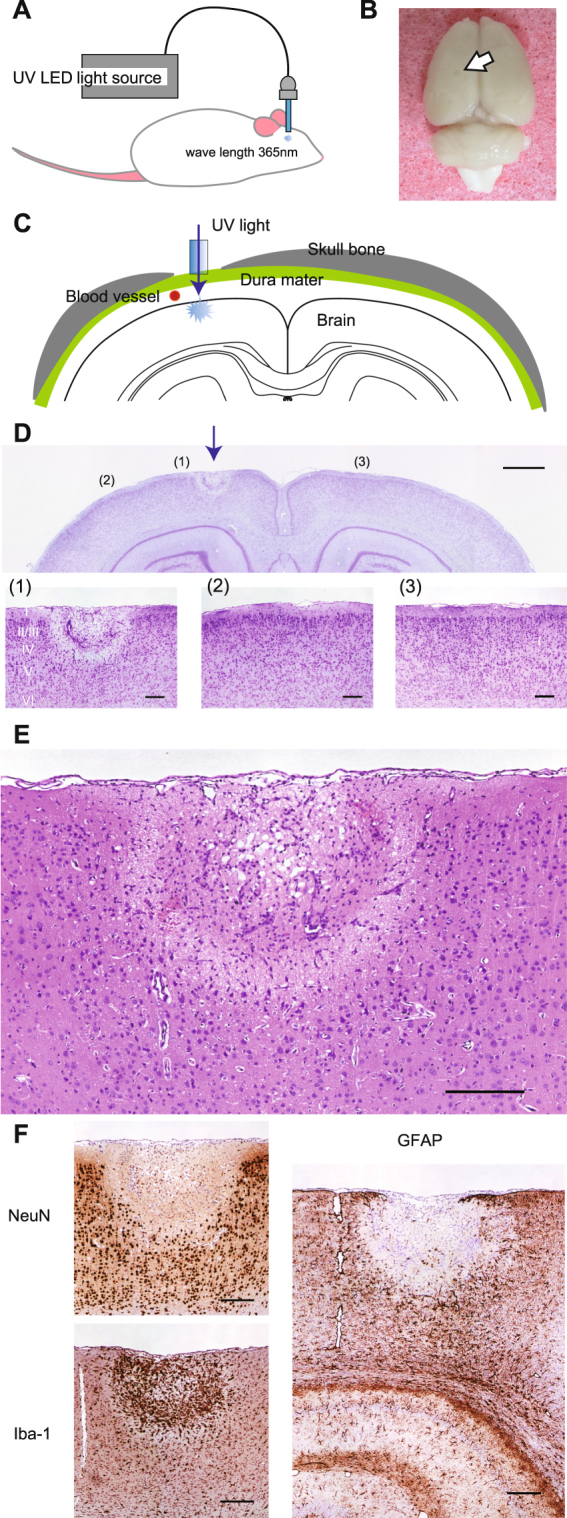
Effects of UV irradiation on the cortical surface above the dura mater. (**A**) Schematic illustration of the apparatus used for UV irradiation. UV light with a wavelength of 365 nm from an LED light source was delivered to the brain of a Wistar rat. (**B**) Round scar imprinted on the brain surface 5 days after UV irradiation. The white arrowhead indicates the site of the scar. (**C**) Schematic illustration of a coronal section of the UV-irradiated rat brain. The optic cannula contacted the dura mater. (**D**) Representative photomicrographs of a Nissl-stained UV-irradiated section (at bregma -3.84 mm). Top: Magnification, x 8. Scale bar, 1000 µm. Blue arrow, site of the optic cannula tip. (1), (2) and (3) indicate corresponding sites in the top and bottom panels. Bottom: Higher-magnification (x 40) photomicrographs of the same brain slice as that in the image in the top panel. (1) UV-irradiated site. (2) Area adjacent to the UV-irradiated site. (3) Area contralateral to the UV-irradiated site. Scale bar, 200 µm. (**E**) Representative photomicrograph of a UV-irradiated section subjected to HE staining. Magnification, x 200. Scale bar, 200 µm. Note that a focal lesion with an inverted bell shape from the brain surface was observed via both Nissl and HE staining. (**F**) Representative photomicrographs of a UV-irradiated section subjected to immunohistochemical staining. The left top, left bottom, and right panels show staining for NeuN (brown, neuronal marker), Iba-1 (brown, microglial marker), and GFAP (brown, astrocyte marker), respectively, and counter-staining with haematoxylin (deep blue). Magnification, x 40. Scale bar, 200 µm. Degeneration of neuronal cells and remarkable congregation of astrocytes and microglial cells were observed at the UV-irradiated site.

First, we investigated whether UV irradiation over the dura mater injures the brain surface in adult Wistar rats. UV light (wavelength 365 nm; power 1.0 mW) emitted from a UV-LED light source was delivered through an optic fibre (Fig. 1A). The skull bone above the target site (approximately 2.0 mm from the midline and 3.7 mm posterior to bregma) was removed, and the tip of the fibre optic cannula (NA = 0.37; core diameter = 400 µm) was placed such that it contacted with dura surface while avoiding major blood vessels (Fig. 1C). UV irradiation of 2.0 mWh (1.0 mW × 2 hours) did not break the dura, and the rats awoke from anaesthesia after closing the incision.

Five days after irradiation, the rats were perfused, and we observed a visible UV irradiation-induced scar on the brain surface (Fig. 1B). Nissl staining revealed an inverted bell-shaped lesion from the brain surface to the upper part of the fifth layer of the cerebral cortex (Fig. 1D, top and bottom-left panels). Notably, the UV irradiation-induced brain lesion (UV-lesion) was confined to the area immediately beneath the cannula tip; no obvious disruption of the cortical layers in an adjacent area (Fig. 1D, bottom-middle panel) or in the side contralateral to the irradiation site (Fig. 1D, bottom-right panel) was observed. Haematoxylin and eosin (HE) staining (Fig. 1E) further revealed degeneration of the acidophilic tissue, a marked decrease in large neuronal cells, accumulation of small immune cells, and appearance of blood vessels inside the lesion. The absence of NeuN immunoreactivity within the lesion, but not in the adjacent and contralateral areas (Fig. S1), indicated loss of neuronal cells in the lesion after UV irradiation (Fig. 1F, top-left panel). GFAP-positive reactive astrocytes were congregated around the lesion (Fig. 1F, right panel). Microglia (Iba-1-immunoreactive cells; Fig. 1F, bottom-left panel), another type of glial cell, were also congregated around the lesion. In contrast to GFAP, Iba-1 immunoreactivity in cells was observed not only around the lesion but also inside the lesion. These findings suggested different roles of these two types of glial cells in tissue subjected to UV irradiation-induced lesioning.

A series of histological observations indicated that UV irradiation of the brain surface, delivered from outside the dura mater, induced the creation of a focal brain lesion. The UV-lesion was characterized by an inverted-bell shape and a sharp boundary between the lesion and adjacent uninjured tissue. Our observations suggested that UV light with a wavelength of 365 nm can induce neuronal cell death by five days after irradiation.

### Little influence of non-UV light irradiation at the same intensity as UV irradiation

**Figure 2:**
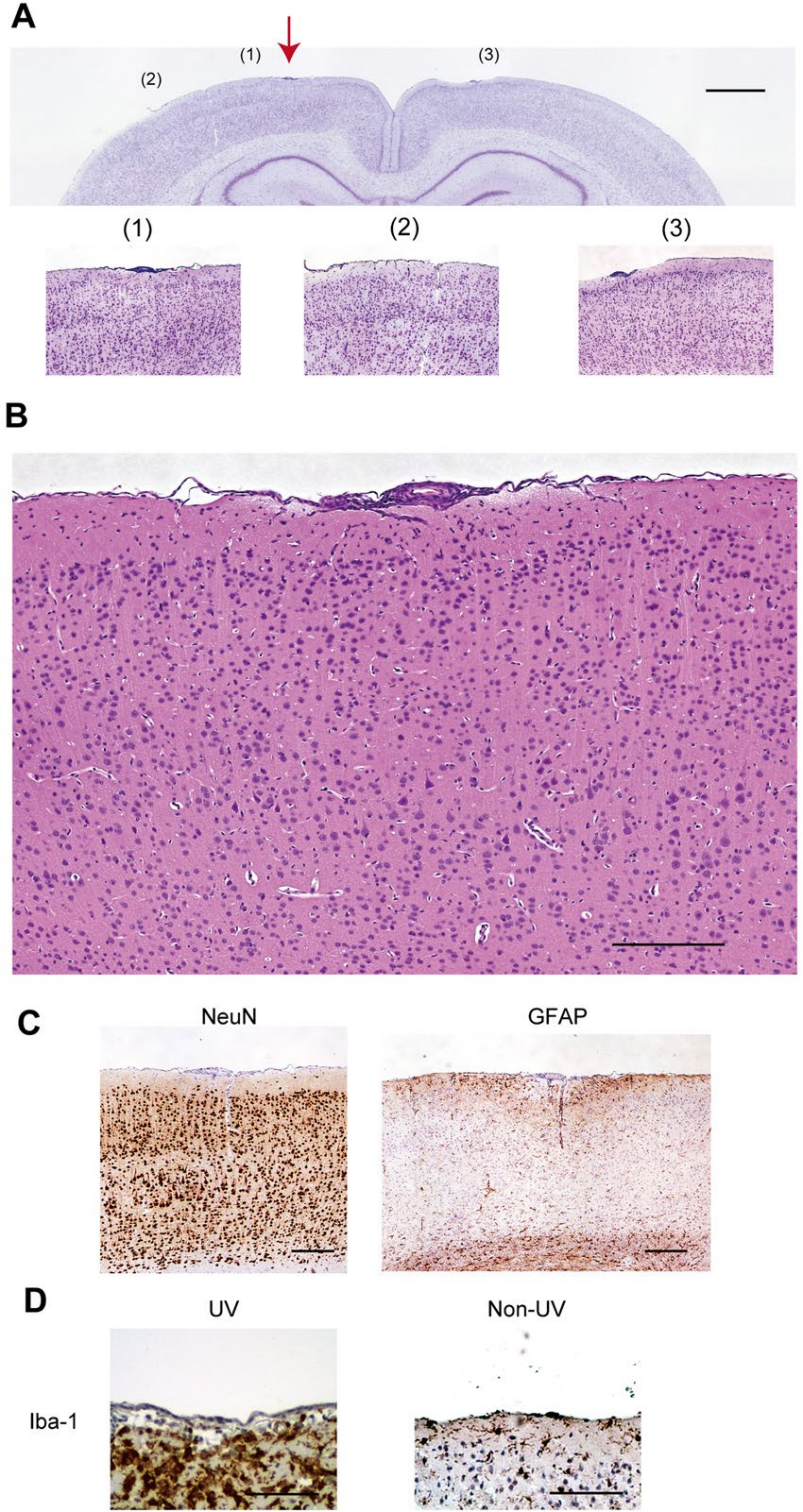
Effects of non-UV irradiation on the cortical surface. (**A**) Representative photomicrographs of non-UV light-irradiated sections subjected to Nissl staining (at bregma −2.92 mm). Top: Magnification, x 8. Scale bar, 1000 µm. Red arrow, site of the optic cannula tip. (1), (2), and (3) indicate corresponding sites on the top and bottom panels. Bottom: Higher-magnification (x 40) photomicrographs of the same brain slice as that shown in the top panel. (1) Non-UV light-irradiated site. (2) Area adjacent to the non-UV light-irradiated site. (3) Area contralateral to the non-UV light-irradiated site. Scale bar, 200 µm. (**B**) Representative photomicrograph of a non-UV light-irradiated section subjected to HE staining. Magnification, x 200. Scale bar, 200 µm. Note that no clear cortical lesion was observed in Nissl or HE staining. (**C**) Representative photomicrographs of non-UV light-irradiated sections subjected to immunohistochemical staining. The left and right panels show staining for NeuN (brown, neuronal marker) and GFAP (brown, astrocyte marker), respectively, and counter-staining with haematoxylin (deep blue). Magnification, x 40. Scale bar, 200 µm. (**D**) Representative photomicrographs of sections subjected to immunohistochemical staining for Iba-1 (brown, microglial marker) and counter-staining with haematoxylin (deep blue). The left panel shows a non-UV light-irradiated section, and the right panel shows a UV-irradiated section. Magnification, x 200. Scale bar, 100 µm. Neuronal degeneration and glial congregation were hardly observed in the sections subjected to non-UV light irradiation.

We found that UV irradiation with a weak intensity of 2.0 mWh delivered from outside the dura induces a focal brain lesion on the cerebral cortex. To investigate whether the induction of the lesion was specific to UV light or a general trait of light across the spectrum, we delivered non-UV light to the brain surface. The non-UV light was produced from a tungsten halogen light source in a broad spectrum of 360 nm~2600 nm. Although this range included UV light (wavelengths shorter than 380 nm), its main spectrum consisted of visible and infrared light. Using the same procedures as described in Fig. 1, 2.0 mWh light from the tungsten halogen light source was applied over the dura, and the brains were sampled five days later. Surprisingly, the non-UV light-irradiated brains did not show any disruption of the cortical layer at the irradiated site (Fig. 2A, top and bottom-left panels; Nissl staining), in the adjacent area (Fig. 2A, bottom-middle panel) or in the contralateral area (Fig. 2A, bottom-right panel). In contrast to the UV-irradiated animals, the non-UV light-irradiated animals showed no destruction of cortical tissue (Fig. 2B; HE-staining) or degeneration of NeuN-immunoreactive cells (Fig. 2C, left). GFAP immunostaining revealed accumulation of a few astrocytes around the irradiated site (Fig. 2C, right); however, the extent of astrocyte accumulation was remarkably smaller than that in the UV-irradiated brains. Similarly, careful observation at high magnification revealed the existence of a few activated microglial cells at the irradiated site (Fig. 2D, left), but the density of activated microglia was far lower than that in the UV-irradiated brain (Fig. 2D, right). These results were replicated 4 times with different animals. These observations suggested that irradiation of non-UV light at an intensity of 2.0 mWh did not induce degeneration of neuronal cells. Although accumulation of some glial cells was observed, the negligible effect of non-UV light irradiation suggested that UV irradiation from outside the dura very efficiently induced the formation of a focal brain lesion on the cerebral cortex.

### Control of the size of the UV-lesion according to the exposure amount

**Figure 3:**
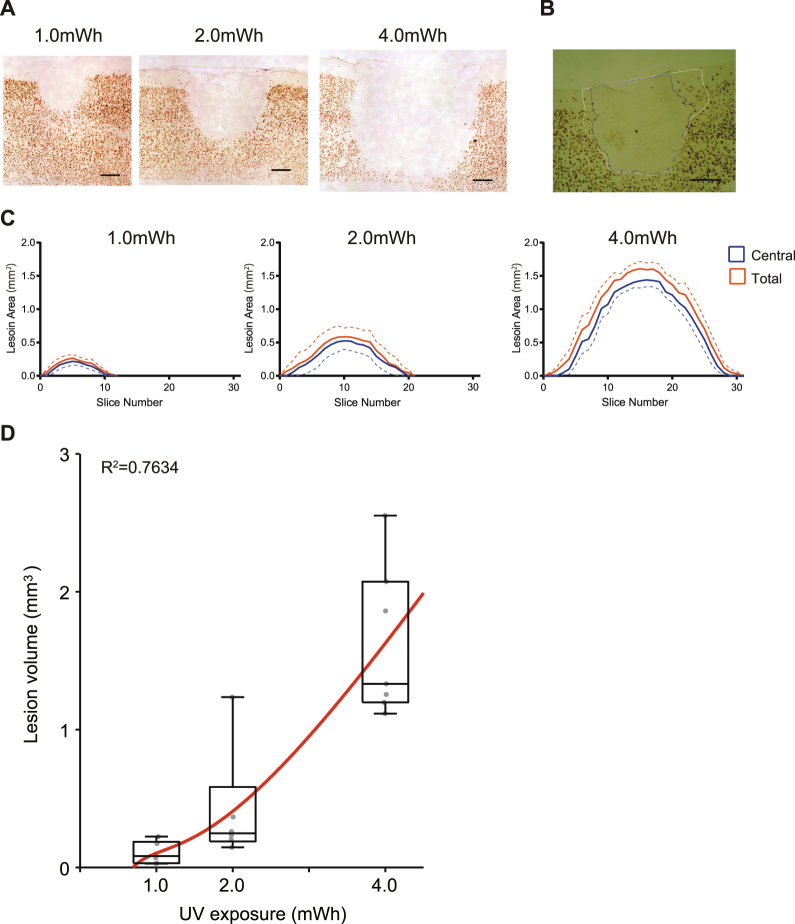
Change in UV-lesion volume depending on the UV light exposure amount. (**A**) Representative photomicrographs of sections exposed to 1.0 mWh (left), 2.0 mWh (middle), or 4.0 mWh (right) UV irradiation subjected to immunohistochemical staining for NeuN. Magnification, x 40. Scale bar, 200 µm. (**B**) Representative photomicrographs of UV-irradiated sections (2.0 mWh), with lesion boundaries drawn using ImageJ for measurement of the lesioned area. The black line indicates the boundary of the central area of the lesion. The white line indicates the boundary of the peripheral area of the lesion. Magnification, x 40. Scale bar, 200 µm. (**C**) The lesioned area calculated from each slice. The average lesioned areas were plotted against the number of slices (N = 11, 20, and 30, for 1.0, 2.0, and 4.0 mWh UV irradiation, respectively). The lesioned area in a slice without a lesion was calculated as 0. Blue lines represent the average central area. Orange lines represent the average total lesioned area (central area + peripheral area). The orange dashed line represents the mean plus SEM, and the blue represents the mean minus SEM. (**D**) The relationship between the total lesion volume and the UV exposure amount. The box plots indicate 25th, 50th, and 75th percentiles as well as maximum and minimum values^39^. The fitted curve (red curve) and raw data (grey dots) were overlaid.

Irradiation with UV light, but not non-UV light, at an exposure amount of 2.0 mWh induced the formation of an inverse bell-shaped lesion on the brain surface. Generally, the effect of light irradiation varies according to not only the wavelength but also the exposure amount. Therefore, we examined the influence of different UV light exposure amounts on lesion formation. As described in Fig. 1, 1.0, 2.0, or 4.0 mWh UV light was applied to the brain surface. Brain slices 30 µm in thickness were divided into 2 series and used for NeuN immunostaining or Nissl staining. NeuN immunostaining revealed visible differences among the three groups in the cross-sectional area at the centre of the lesion (Fig. 3A). In contrast to the 2.0 mWh UV irradiation group, with lesions to the depth of layer 5 (Fig. 3A, middle panel), the 1.0 mWh UV irradiation group had lesions that reached layer 4 (Fig. 3A, left panel). Furthermore, 4.0 mWh UV irradiation induced a remarkably larger lesion that penetrated all 6 layers of the cerebral cortex (Fig. 3A, right panel). Notably, changing the exposure amount did not change the shape of the lesion, in contrast to its effect on the lesion volume. To evaluate the lesion volume quantitatively, the boundary of the lesion was defined in each NeuN-immunostained slice (Fig. 3B). The lesion area was categorized into a central area without NeuN-immunoreactive cells (Fig. 3B, enclosed by the blue line) and a peripheral area with sparse NeuN-immunoreactive cells (Fig. 3B, enclosed by the white line). The peripheral and central areas were measured in each brain slice and plotted from anterior to posterior (Fig. 3C). The central area (blue lines) occupied most of the total lesion area (orange lines), corresponding to the finding that the boundary of the UV-lesion was sharp. The number of slices was 8.17 ± 2.14 in the 1.0 mWh UV irradiation group (n = 6; left panel), 13.67 ± 3.72 in the 2.0 mWh UV irradiation group (n = 6; middle panel), and 24.71 ± 3.82 in the 4.0 mWh UV irradiation group (n = 7; right panel). A statistical analysis of the number of slices constituting the lesion area revealed a significant main effect of light exposure amount (Kruskal-Wallis test, H2 = 15.13, p < 0.0001). Comparison of the lesion volume revealed that exposure to a larger amount of UV light induced the formation of a larger lesion in the cerebral cortex (Fig. 3D; Kruskal-Wallis test, H2 = 14.12, p < 0.0001). The total lesion volume was well approximated by a cubic logarithmic function (${\rm{y}}=0.4579\,{(ln(x))}^{3}+0.2186\,{(ln(x))}^{1}+0.1036$, red curve in Fig. 3D, r^2^ = 0.7634).

### Confirmation of the UV-lesion area in a living animal with magnetic resonance imaging (MRI)

Except for the case of histological studies on lesioned tissue, a focal brain lesion is created to examine its influence on a living animal. Thus, it is necessary for an experimenter to know whether the lesion was successfully created at the targeted site *in vivo*. Based on the above experiments, we confirmed the UV-lesion site histologically using samples from postmortem brains. However, confirming the creation of the UV-lesion at the targeted site in a living brain is critical for experiments using awake animals.

**Figure 4:**
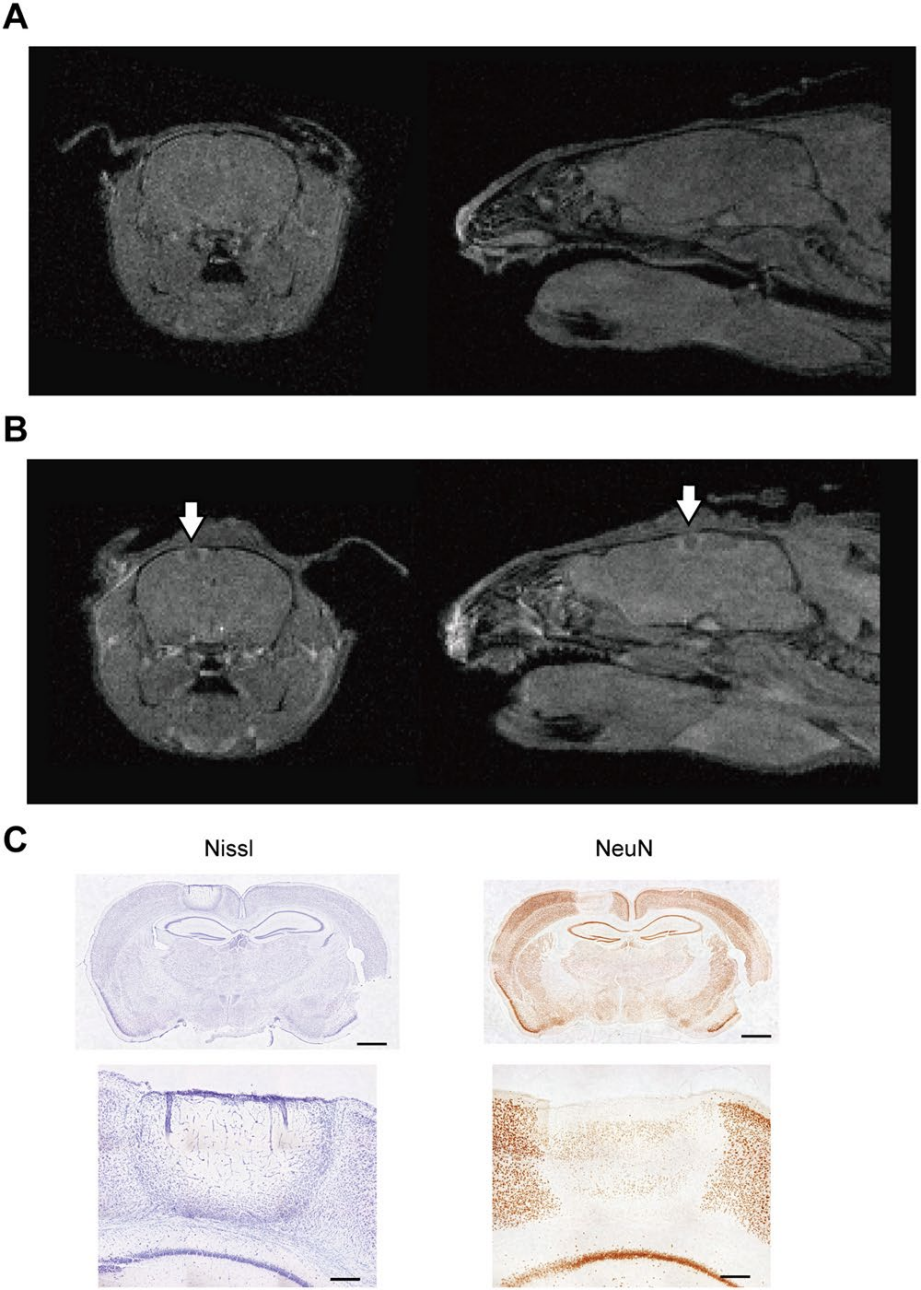
MRI for visualization of the UV-lesion in a living animal. (**A**) MEMR images (with a T1-weighted three-dimensional fast low-angle shot (3D-FLASH) sequence) of the mouse brain before UV irradiation. Coronal (left) and sagittal (right) images showing no injury. (**B**) MEMR images with a T1-weighted 3D-FLASH sequence of the same mouse brain as in (A), 5 days after 2.0 mWh UV irradiation. The white arrows indicate the location of UV irradiation. Coronal (left) and sagittal (right) images demonstrating that the UV-lesion can be visualized in a living animal using MRI. (**C**) Representative photomicrographs of UV-irradiated sections of the mouse brain. The left and right panels show Nissl staining and immunohistochemical staining for NeuN, respectively, of sections from the same mouse as in A and B (at bregma −2.18 mm). Top panels: magnification, x 8. Scale bar, 1000 µm. Bottom panels: magnification, x 40. Scale bar, 200 µm.

To visualize the effect of the UV-lesion in a living brain, MR images were compared between before and after UV irradiation. Manganese chloride (MnCl_2_) was systemically injected into adult ICR/Jcl mice (n = 6) one day before the first imaging for MRI enhancement^16–18^. Five days after UV irradiation, a low-intensity area was observed at the UV-irradiated site in both coronal (Fig. 4B, left panel) and sagittal (Fig. 4B, right panel) images compared to the images before UV irradiation (Fig. 4A). Since injected MnCl_2_ is known to stain cells for several days^18,19^, a low-intensity area on the brain surface should indicate destruction of the cells after UV irradiation. Histological analysis after the final imaging further confirmed the existence of the lesion, in agreement with the MRI findings (Fig. 4C). These results were replicated 6 times with different animals. Taken together, we succeeded in visualizing UV irradiation-induced degeneration of the brain tissue in living animals using manganese-enhanced MRI (MEMRI).

### Elimination of neural activity around the UV-lesion site

Our UV-lesion technique will be useful for investigating the effect of focal elimination of neural activation in a brain region of interest. Thus, we examined whether neural activity evoked by electrical stimulation was altered at the UV-irradiated site using voltage-sensitive dye (VSD) imaging.

**Figure 5:**
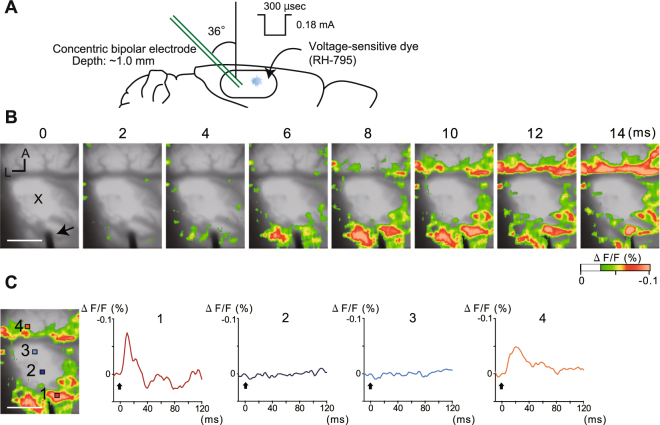
Optical imaging of the spread of neural activation evoked by electrical stimulation adjacent to the UV-irradiated site. (**A**) Schematic illustration of electrical stimulation and VSD imaging. (**B**) Spatiotemporal dynamics of evoked neural activity after single-pulse stimulation from the stimulation electrode (black arrow on the bottom of the image). The electrical stimulus was applied at 0 ms. The VSD signal was colour-coded and superimposed on the background cortical image. X indicates the UV-irradiated site. Scale bar, 1000 µm. (**C**) Representative time course of the VSD signals at 4 points in or near the UV-irradiated site. Black arrows (time 0) indicate the time point of electrical stimulation. Each trace shows the signal in the pixel area, indicated by the respective coloured rectangles in the left panel (12 ms after stimulation).

Five days after 2.0 mWh UV irradiation, the cortical surface was stained with the VSD, and electrical stimulation was applied with a stimulation electrode inserted into the cerebral cortex at 2~5 mm posterior to the irradiated site (Fig. 5A). Single-pulse cortical stimulation evoked neural activity. A decrease in fluorescence intensity indicating depolarization was observed around the electrode in the initial milliseconds after stimulation (Fig. 5B). Thereafter, the depolarizing response spread throughout the field of view, except for the UV-irradiated site, indicated as “X” on Fig. 5B. Figure 5C shows the temporal change in the VSD signal at 4 points in the vicinity of the electrode. In addition to point 1, point 4 on the other side of the lesion showed a sharp increase in the VSD signal after stimulation. However, no change in the VSD signal was observed at points 2 and 3, which were located inside the UV-irradiated site. These results were replicated 3 times with different animals. These results demonstrated that 2.0 mWh UV irradiation eliminated neural activity after electrical stimulation in an area adjacent to the irradiated site.

## Discussion

In the present study, we developed a novel experimental method to create a focal brain lesion on the cortical surface by irradiation with UV light. This UV-lesion method is less invasive than traditional lesion techniques since UV irradiation induced neuronal degeneration without breaking the dura mater, as observed on MRI without sacrificing the animal. Moreover, we succeeded in controlling the lesion volume by changing the UV light exposure amount. Confirmation of the elimination of neural activation at the UV-irradiated site further indicated that the UV-lesion method will be useful for a variety of lesion experiments.

We used 1.0~4.0 mWh UV irradiation at a wavelength of 365 nm to create a focal brain lesion. By contrast, irradiation with 2.0 mWh non-UV light from a halogen light source failed to create a lesion. Previously, however, Suzuki *et al*.^20^ reported that 2.5 minutes of irradiation with light from a 90-W halogen bulb (i.e., exposure amount of 3750 mWh) induced “photo injury” in a mouse brain. This thousand-fold difference in exposure amount between studies suggested that visible light may be able to induce brain injury, but its effective dose was much higher than that of UV light. The large difference in lesion efficacy between visible and UV light also suggests that the molecular mechanisms underlying neuronal death following photo-injury and our UV-lesion are unlikely to be the same. How UV irradiation used in the UV-lesion method induces neuronal death should be further investigated. It is known from previous studies, mainly studies of skin, that most of the cellular damage caused by UV-A light (including 365 nm UV light) is due to ROS production by organelles including mitochondria^12^. When cellular and/or genomic damage by ROS and UV light itself accumulates beyond a threshold, the molecular process of programmed cell death is initiated^21,22^. Such a deterministic property with a threshold of damage accumulation may be the reason for why we easily determined the boundary between the UV-lesion and intact tissue in the NeuN-immunostained slices.

In addition to neuronal loss, we observed accumulation of the glial cells at the UV-irradiated site. Five days after UV irradiation, astrocytes began to migrate from the intact tissue and gathered around the UV-irradiated site. We also observed microglial cell accumulation inside the lesion. These reactions to UV irradiation followed a comparable time course to that described in a previous report, in which glial cells accumulated at 4 days after the photo injury, with enormous amount of non-UV light irradiation^20^. Generally, astrocytes react to a pathological state of brain tissue in a process of reactive astrogliosis, in which astrocytes prevent the outflow of potentially deleterious debris by forming a barrier around the lesion^23^. Additionally, activated microglia remove this debris and promote tissue repair inside the lesion^24^. It would be intriguing to investigate whether the UV-irradiated site follows a similar long-term recovery process, including gliosis, to that reported previously^20,25,26^.

In the present study, astrocyte accumulation was observed not only via histological analysis but also on MEMRI. Careful examination of the MR images after irradiation (Fig. 4B) revealed that there was a high-intensity (white) area around the lesioned site, which had lower signal intensity. Mn^2+^ within the extracellular fluid of the brain is known to be transported into neurons and astrocytes through divalent metal transporter 1 (DMT 1)^27–29^. It was reported that 80% of brain manganese is stored in glutamine synthetase, which is mainly expressed in astrocytes, although it is also expressed in neurons^30,31^. These findings explain the high MR signal intensity in the area surrounding the lesion, which may reflect accumulation of astrocytes. Conversely, very low MR signal intensity inside the lesion, in which microglia congregated, may reflect the absence of neurons and astrocytes. It was also suggested that the recovery process after UV irradiation, represented by astrocyte accumulation, can be continuously observed in the same living animal using MEMRI as previously reported with other lesion methods^16,18^.

The lesions induced with 4.0 mWh UV irradiation were not only deeper but also wider than the lesions induced with 1.0 and 2.0 mWh UV irradiation. UV light spreads from the centre of the optic cannula because of diffraction. As the exposure amount increased, the lesion became larger while maintaining a similar shape. This result may be because the area receiving an amount of UV light that exceeded the threshold for cell death became wider with larger amounts of UV irradiation. Thus, the lesion volume increases with increasing exposure amount, as demonstrated in the present study. The lesion volume was well fitted under the assumption of a cell death threshold. However, if the amount of UV exposure is sufficiently large, the lesion volume may be saturated because of the limited permeability of UV light.

We applied UV irradiation at a constant dose rate (1.0 mW/h) throughout the experiments. The effect of UV irradiation should be influenced not only by the exposure amount but also the dose rate. Additionally, fractionated irradiation is known to attenuate radiation-induced damage^32^. In the present study, we demonstrated that UV irradiation for a prolonged duration induced neuronal death even when the dose rate was relatively small, and the UV light was delivered with a UV-LED light source used for optogenetic stimulation. The application of UV light for optogenetic experiments has been developed recently^33^. However, the relationship between the conditions of UV irradiation, including the exposure amount and dose rate, and the size and occurrence of a lesion have been less comprehensively studied. Therefore, these investigations will be useful not only for the application of the UV-lesion model but also for optogenetic studies without producing any artefacts of UV irradiation-induced cellular damage.

In the present study, we confirmed eliminated neural activation at the site of the UV-lesion using optical imaging. Stimulation-evoked activation arrived at the opposite side of the lesion (location # 4 in Fig. 5C) by proceeding across the lesion. This observation suggested that the signal transmission ability of NeuN-immunoreactive cells around the lesion was not destroyed. Therefore, we concluded that the UV irradiation technique produced a lesioned area with defined borders in terms of not only morphology but also function. By changing the size, depth, and shape of the lesion according to the UV light exposure amount, our method may be useful for investigating the mechanisms and structures of local connections in the cortex^34^. Additionally, UV irradiation to produce a lesion with a desired shape and size in a cultured brain slice would enable us to ablate specific neural circuits of interest. These features will allow us to apply the UV-lesion method to electrophysiological studies.

There are some limitations to our UV lesion method. First, UV irradiation to brain regions other than the dorsal cortex, where the optic canula cannot be accessed vertically, is not easy. Modifying the tip of the optic cannula may widen the target cortical areas. Second, we removed the skull over the targeted site for UV light penetration. Development of less invasive techniques to create viewing windows through the skull (e.g., skull thinning) should be further considered in future applications of the UV lesion method. Additionally, UV irradiation induced non-specific cellular damage under the given conditions. The possibility of a cell-type specific influence of UV irradiation should be further investigated in future studies.

This UV-lesion method has been developed for creating a focal brain lesion in a less invasive and more easily controllable manner than traditional lesioning methods. This method may be widely applicable since the volume of the UV-lesion can be easily estimated. Theoretically, the shape of the UV-lesion could also be modulated by adjusting the shape and size of the optic cannula tip. Moreover, our method does not require any specific equipment or drugs other than a commercially available UV-LED light source used for optogenetics. Taken together, the UV-lesion method can be applied not only to neuroscientific experiments but also to pathological investigations of focal brain injury because of its controllability and simplicity.

## Methods

### Experimental animals

All procedures were conducted in accordance with the National Institutes of Health guidelines and were approved by the Animal Care and Use Committee of the National Institute of Advanced Industrial Science and Technology (AIST). All efforts were made to minimize the number of animals and their suffering.

Adult male Wistar rats (total of n = 30; 311.93 ± 48.93 g BW at the time of irradiation; purchased from SLC Japan Inc., Japan) and adult male ICR/Jcl mice (n = 6 for MRI; 37.12 ± 3.76 g BW at the time of irradiation; purchased from CLEA Japan Inc., Japan) were used. All animals were housed under standard conditions (12 h light/dark cycle). Food and water were provided *ad libitum*.

### Irradiation with UV or non-UV light

Animals were anaesthetized with an injection of ketamine (80 mg/kg, i.m.) and xylazine (10 mg/kg, i.p.) and placed in a stereotaxic frame (Narishige, Inc., Tokyo, Japan). A constant level of anaesthesia was maintained with 1% isoflurane. After removal of the skull bone, either 1.0 mW UV light (measured at the tip of the optic cannula using a power mater, PM100D console with S130VC sensor, Thorlabs, Inc., Newton, USA; 365 nm wavelength) from a UV-LED light source (LEDFLP-1CH_500, Doric Lenses, Inc., Quebec, Canada) or non-UV light from a halogen light source (SLS201L/M, Thorlabs, Inc., Newton, USA) was delivered through an optic cannula (MFC_400/430-0.37_12 mm_SM3_FLT, Doric Lenses, Inc., Quebec, Canada, 400 μm core diameter, 0.37 NA) contacting dura mater while avoiding blood vessels under the dura (for histological analyses in rats, anteroposterior (AP) -3.0~4.0 mm from bregma, mediolateral (ML) 1.5~2.5 from the midline; for VSD imaging, AP -3.5~4.0, ML 2.5~3.5; for MRI in mice, AP -2.0 ~3.0, ML 1.5~2.5). Animals received unilateral irradiation and were exposed to 2.0 mWh UV (n = 5) or non-UV light (n = 4) for qualitative histological analysis or 2.0 mWh UV light for MRI (mice, n = 6) and VSD imaging (n = 3). For lesion volume measurement, each rat received bilateral or unilateral irradiation with 1.0, 2.0, or 4.0 mWh UV light (n = 16). The remaining 2 rats were used for examination of the effect of blood vessel irradiation (see Discussion and Fig. S2). The animals were transcardially perfused 5 days after irradiation, and brain samples were processed for subsequent histological analyses.

### Preparation of brain tissues for histological analysis

For qualitative histological analysis, coronal sections of paraffin-embedded brain slices 4 µm in thickness were generated using a sliding microtome. Then, the sections were deparaffinized and subjected to Nissl staining with 0.1% cresyl violet solution, HE staining, or immunohistochemistry.

Alternatively, brain tissues used for lesion volume measurement and tissues collected after MRI and VSD imaging were embedded with 10% gelatine solution in 0.1 M PB. After post-fixation with 4% paraformaldehyde in 0.1 M PB and cryoprotection, coronal sections 30 µm in thickness were prepared using a freezing microtome. Serial sections were collected in two sets with 60-µm intervals. One of the sets was processed for Nissl staining with 0.2% thionin blue solution, and the other was processed for free-floating immunohistochemistry for NeuN.

### Immunohistochemistry for qualitative histological analysis

Immunohistochemical staining for NeuN and GFAP was performed using a Bond-Max automated immunohistochemical staining machine (Leica Microsystems, Milton Keynes, UK). The primary antibodies used were anti-NeuN (clone: A60, 1:100; Merck Millipore, Billerica, USA) and anti-GFAP (clone: 6F2, 1:200; DAKO A/S, Glostrup, Denmark).

Immunohistochemical staining for Iba-I was performed as follows: Deparaffinized sections on silane-coated glass slides were incubated in 0.02 M Tris-HCl buffer, pH 9.0, for 30 min at 90 °C. After cooling at room temperature (RT) and washing, the sections were incubated in methanol with 3% H_2_O_2_ for 20 min at RT for blocking of endogenous peroxidase (POD) activity. After washing, the sections were pretreated with PBS containing 0.2% Triton X (PBS–X) and 4% Block-Ace (blocking buffer; DS Pharma Biomedical Co., Ltd., Osaka, Japan) for 2 hours at RT. The sections were then incubated with goat polyclonal anti-Iba-1 antiserum (1:100; ab104225, Abcam plc, Cambridge, UK) in blocking buffer for one night at RT. Then, the sections were washed and incubated with biotinylated horse anti-goat secondary antiserum (1:250; BA-5000; Vector Laboratories, Inc., Burlingame, USA) for 2 hours at RT. After washing, the sections were reacted with avidin-biotin complexes (VECTASTAIN ABC Standardkit, PK-4000; Vector Laboratories, Inc., Burlingame, USA) in PBS for 1 hour at RT and then washed. Next, the sections were incubated in 0.02% diaminobenzidine (DAB) and 0.003% H_2_O_2_ in 0.05 M Tris-HCl buffer (pH 7.5) containing 0.01 M imidazole for 5 min, followed by washing with PBS. All sections were counter-stained with haematoxylin, dehydrated through an ascending ethanol series, cleaned with xylene, and coverslipped with Permount (Thermo Fisher Scientific Inc., Waltham, USA).

### Free-floating immunohistochemistry for NeuN

Freely floating sections were incubated in PBS-X with 0.3% H_2_O_2_ for 20 min at RT for blocking of POD activity. After washing, the sections were pretreated with 3% normal goat serum in PBS–X (blocking buffer) for 2 hours at RT. The sections were then incubated with rabbit polyclonal anti-NeuN antiserum (1:2,000; ab104225, Abcam plc, Cambridge, UK) in blocking buffer for one night at 4 °C. Next, the sections were washed and incubated with biotinylated goat anti-rabbit secondary antiserum (1:250; BA-1000; Vector Laboratories, Inc., Burlingame, USA) for 2 hours at RT. After washing, sections were reacted with avidin-biotin complexes (VECTASTAIN ABC Elite kit; Vector Laboratories, Inc., Burlingame, USA) in PBS for 1 hour at RT and then washed. The sections were subsequently incubated in 0.02% DAB and 0.003% H_2_O_2_ in PBS for 3 min, followed by washing with PBS. All sections were mounted on MAS-coated slides (Matsunami Glass Ind., Ltd., Osaka, Japan), air-dried, dehydrated through an ascending ethanol series, cleaned with xylene, and coverslipped with Permount (Thermo Fisher Scientific Inc., Waltham, USA).

### Measurement of the lesion volume

The UV-lesion volume was measured using NeuN-stained slices. Four samples were excluded from the analysis because of possible blood vessel irradiation (see Discussion and Fig. S1). The area without NeuN-immunoreactive (ir) cells was defined as the central area, and the area with sparse NeuN-ir cells was defined as the peripheral area. Whether each slice contains a lesion area was judged from observation of adjacent Nissl-stained sections. An outline of the lesion was manually traced, and the area was calculated using the image processing software ImageJ (National Institutes of Health, Bethesda, USA). The lesion volume was calculated using following formula with an assumption that the lesion area of adjacent slices to the first and last lesioned slice was 0 µm^2^: Lesion volume (µm^3^) = Σ(lesion area in each slice (µm^2^)) × 60 (µm). The number of lesioned slices and the lesion volume were analysed with the Kruskal-Wallis test for the main effect of light exposure amount. The statistical analyses were conducted with GraphPad PRISM 7 (GraphPad Software, Inc., San Diego, USA).

During the experiment, we observed a few “abnormal” lesions. Unlike a “normal” UV-lesion with an inverted-bell shape and obvious boundary, these abnormal lesions showed the following features: ambiguous boundary with very low NeuN-immunoreactivity around the lesion, triquetrous shape, very large size, and/or heavy bleeding inside the lesion. Because accurate measurement of the lesion area was not possible, four abnormal lesions were excluded from the analysis. We hypothesised that the positional relationship between the cannula tip and blood vessels under the dura may affect the occurrence of those “abnormal” lesions (Fig. S2).

### Fitting of the lesion volume

The effect of UV irradiation on a tissue can be assumed to decrease in an exponential manner because of dampening of the UV light in brain tissue, namely,

1 $$\tau {I}_{0}\cdot {{\rm{e}}}^{-{\rm{\alpha }}\cdot {\rm{r}}}\le {\rm{W}}$$

where *τ*, *I*_0_, *α*, *r*, and *W* denote the irradiation duration, the effect per unit of time on the tissue just below the tip of the optic fibre, the decay coefficient, the distance from the tip of the optic fibre, and the assumed UV light exposure threshold for cell death, respectively. Thus, the distance in which the lesion occurs can be described as

2 $${\rm{r}}\le \frac{1}{\alpha }ln(\tau )+Const.$$

Since the light spreads in the 3-dimensional space, we fitted the volume using the following equation with 4 parameters (*a*_0_ − *a*_3_).

3 $${\rm{V}}={a}_{3}{(ln(\tau ))}^{3}+{a}_{2}{(ln(\tau ))}^{2}+{a}_{1}{(ln(\tau ))}^{1}+{a}_{0}$$

The fitting was conducted with MATLAB (MathWorks, Inc., Natick, USA).

### *In vivo* MEMRI

One day before the day of the first MRI and UV irradiation, mice (n = 6) received i.p. injection of 50 mM MnCl_2_ (62.92 mg/kg). UV irradiation was conducted using the same procedures as described above. MR images were obtained twice: preceding and 5 days after irradiation. The mice were anaesthetized with 2.0% isoflurane and fixed on a polymethylmethacrylate holder in the prone position. The mice underwent MRI using the MR scanner (MRmini SA1506, DS Pharma Biomedical Co., Ltd., Osaka, Japan) equipped with a 1.5-T permanent magnet and a solenoid MRI coil (30 mm inner diameter). Coronal and sagittal MR images were obtained using a T1-weighted three-dimensional fast low-angle shot (3D FLASH) sequence. The imaging parameters for MEMRI were set as follows: repetition time (TR) = 50 ms, echo time (TE) = 4.15 ms (coronal) or 3.6 ms (sagittal), flip angle (FA) = 90°, field of view (FOV) = 20 × 40 × 40 mm, in-plane matrix = 256 × 128, slice thickness = 0.3125 mm (128 coronal slices) or 0.625 mm (64 sagittal slices), and number of excitations (NEX) = 2. After completion of MRI on the fifth day after UV irradiation, the mice were transcardially perfused, and brain samples were processed for Nissl staining and NeuN immunostaining.

### VSD imaging

Neural activity around the lesioned site evoked by electrical stimulation was investigated using VSD imaging^35–38^. Five days after 2.0 mWh UV irradiation, adult male Wistar rats (n = 3) were anaesthetized with an injection of ketamine (80 mg/kg, i.m.) and xylazine (10 mg/kg, i.p.) and placed in a stereotaxic frame. After removal of the skull bone (AP 6 mm and ML 3 mm around the irradiated site) and the dura, the exposed cortex was stained for 1 h with the VSD RH-795 (Life Technologies, Carlsbad, USA). A concentric bipolar electrode (IMB-9002; Inter Medical, Nagoya, Japan) was inserted into the cerebral cortex in the vicinity of the lesion site (2~5 mm anterior to the centre of the irradiated site) with an insertion angle of 36° to the posterior and a depth of ~1.0 mm. Neural activity was recorded as fractional changes in fluorescence (ΔF/F) using a Micam01 system (Brainvision, Inc., Tokyo, Japan). The fluorescence signals were captured with Micam01-CCD camera (BrainVision, Inc.) with 88 × 60 pixels at a 500-Hz frame rate covering an area of approximately 2.9 × 2.2 mm. In each trial, single-pulse electrical stimulation (300 µs in duration and 180 µA in intensity) was applied, and the average VSD signals of 12 consecutive trials with 12 sec intervals were obtained.

### Data availability

The datasets analysed during the current study are available from the corresponding authors upon reasonable request.

## Electronic supplementary material

Supplementary figures
