# Supplementary material for: Focal brain lesions induced with ultraviolet irradiation
Source: Sci Rep. 2018 May 22;8:7968. doi: 10.1038/s41598-018-26117-w (PMC5964180; doi:10.1038/s41598-018-26117-w)
Supplement: Supplementary file 1 — Supplementary figures [file 41598_2018_26117_MOESM1_ESM.pdf]

Focal brain lesions induced with ultraviolet irradiation

Mariko Nakata[1,2,*], Kazuaki Nagasaka[1,2,3], Masayuki Shimoda[4], Ichiro Takashima[1,3], Shinya Yamamoto [1,*]

[1] Systems Neuroscience Group, Human Informatics Research Institute, National Institute of Advanced Industrial Science and Technology (AIST), 1-1-1 Umezono, Tsukuba 305-8568, Japan

[2] Research Fellow, Japan Society for Promotion of Science, 5-3-1 Koujimachi, Chiyoda-ku, Tokyo 102-0083, Japan

[3] Graduate School of Comprehensive Human Sciences, University of Tsukuba, 1-1-1 Tennodai, Tsukuba 305-9577, Japan

[4] Department of Pathology, Keio University School of Medicine, 35 Shinanomachi, Shinjuku-ku, Tokyo 160-8582, Japan

* nakata-m@aist.go.jp

* yamamoto-s@aist.go.jp

Supplemental Figures

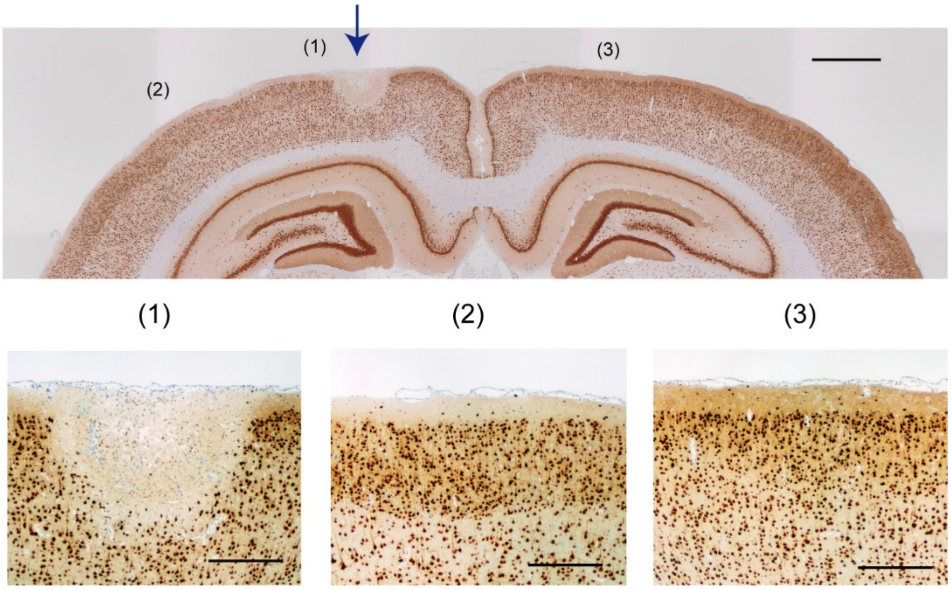

Figure S1. Representative photomicrographs of a UV-irradiated section subjected to immunohistochemical staining for NeuN (at bregma -3.84 mm). Top: Magnification, x 8. Scale bar, 1000 µm. Blue arrow, site of the optic cannula tip. (1), (2) and (3) indicate corresponding sites on the top and bottom panels. Bottom: Higher-magnification (x 40) photomicrographs of the same brain slice as that in the image in the top panel. (1) UV-irradiated site. (2) Area adjacent to the UV-irradiated site. (3) Area contralateral to the UV-irradiated site. Scale bar, 200 µm. Undisrupted NeuN expression was observed in the areas adjacent and contralateral to the lesion.

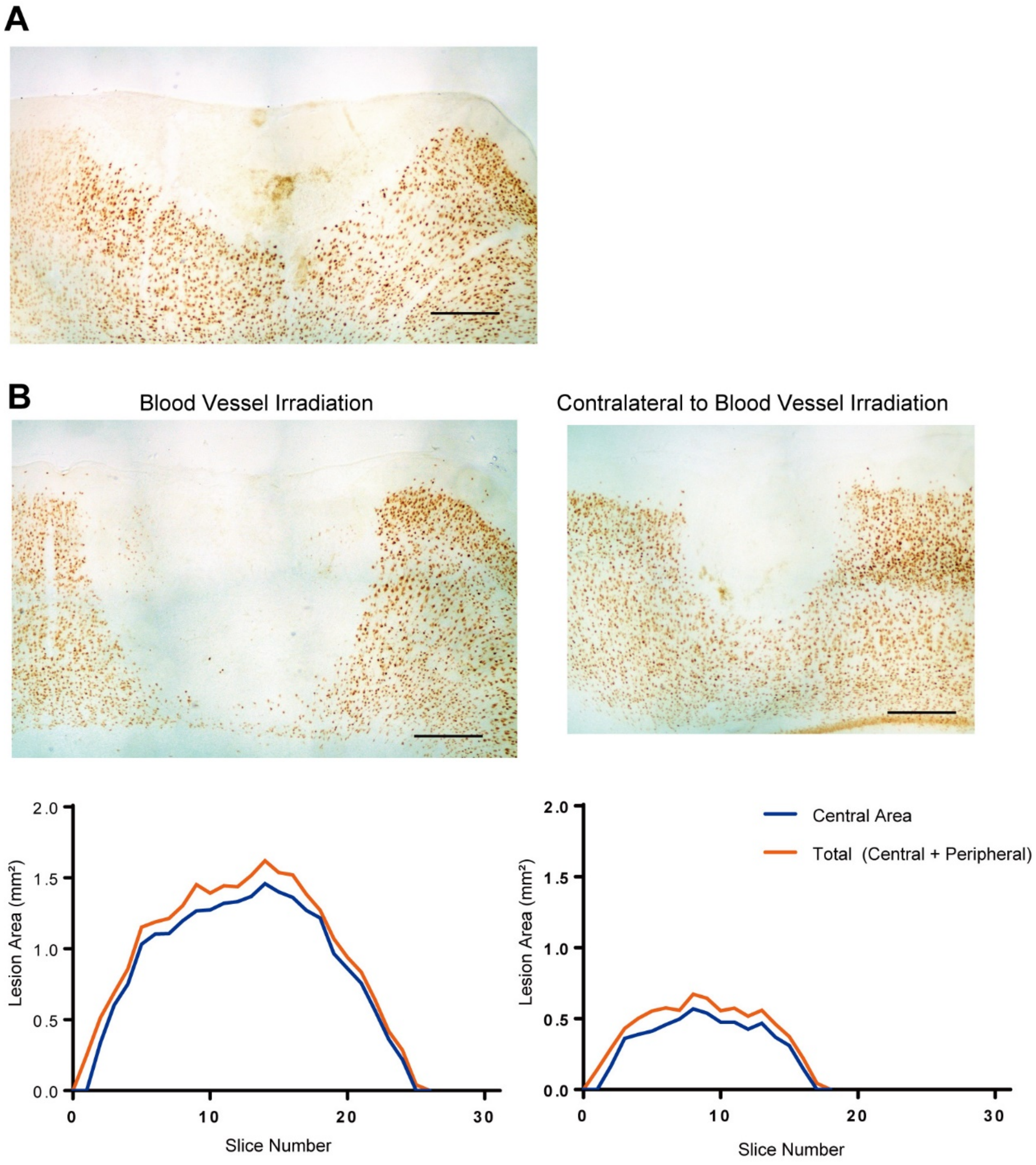

Figure S2. Possible effects of blood vessel irradiation on lesion size and shape. **(A)** Representative photomicrographs of a section exposed to 0.33 mWh (1.0 mW x 20 min) UV irradiation on a blood vessel subjected to immunohistochemical staining for NeuN. The cannula tip was placed over a thick blood vessel. A lesion with a very large size and triquetrous shape was observed, and bleeding inside the lesion was detected. **(B)** To examine the effect of blood vessel irradiation, a rat received 2.0 mWh UV irradiation bilaterally. One side, the cannula tip was placed over a thick blood vessel (left panels, blood vessel irradiation). On the other side, UV irradiation was applied to a site that avoided blood vessels (right panels, no blood vessel irradiation). Top panels, representative photomicrographs of

sections of each hemisphere subjected to immunohistochemical staining for NeuN. Left, UV light was applied over a blood vessel. Right, section without blood vessel irradiation showing the area contralateral to that in the left panel. Bottom panels, lesion area calculated from each slice. The lesioned areas determined by the boundaries were plotted against the number of slices (N = 25 and 17 for the left and right panels, respectively). Blue lines represent the average central area. Orange lines represent the average total lesion area (central area + peripheral area). A larger lesion was observed on the side with blood vessel irradiation than on the opposite side. **(A)** and **(B)** Magnification, x 40. Scale bar, 200 µm. Note that UV irradiation of a thick blood vessel induced a triquetrous and/or an extremely large lesion, which were the features of “abnormal” lesions (see Methods). It was suggested that UV irradiation of a thick blood vessel may induce a different, specific pattern of brain injury. The influence of irradiating UV onto blood vessels should be investigated more in detail in future studies.
